# On-site medical interventions for spectators at venues during major international sporting tournaments: a prospective comparative study of four single-sport and multi-sport tournaments

**DOI:** 10.3389/fpubh.2026.1812763

**Published:** 2026-05-07

**Authors:** Elżbieta Lipska, Marzena Mikrut, Agata Pawlak

**Affiliations:** 1 Institute of Mother and Child, Warsaw, Poland; 2 2nd Instance Polish Antidoping Hearing Panel, Warsaw, Poland; 3 University Hospital, Wrocław, Poland; 4 Polish Medical Air Rescue, Warsaw, Poland

**Keywords:** emergency response planning, health management, major sporting tournament, mass gathering medicine, on-site medical intervention, prehospital medical services

## Abstract

**Introduction:**

Major sporting tournaments (MSTs) are complex mass-gathering events that place demands on local medical services. Although single-sport (SS-MSTs) and multi-sport tournaments (MS-MSTs) share core planning principles, differences in event structure may influence medical service utilization.

**Objective:**

This study compared on-site medical utilization, and hospital transport rates across SS-MSTs and MS-MSTs, emphasizing evidence-based insights to guide scalable and effective medical staffing.

**Methods:**

A prospective analysis was performed using daily reported medical data from four MSTs: UEFA EURO 2012, EHF EURO 2016 (SS-MSTs), The World Games 2017 and the European Games 2023 (MS-MSTs). Patient presentations (PPs), transports to hospital (TTHs), and medical-event-to-transport-to-hospital rates (METH) were analyzed. PP-rates (PPRs) and TTH-rates (TTHRs) were calculated per 10,000 attendees. Comparisons were conducted between tournaments and tournament types.

**Results:**

Across 2,188,497 spectators, 1,539 PPs and 135 TTHs were recorded. Spectator PPRs were higher at SS-MSTs than at MS-MSTs (5.71 vs. 3.97), while TTHRs (0.42 vs. 0.48) and METH (6.09 vs. 10.08) were similar. Fewer than 10% of on-site presentations required hospital transport. SS-MSTs exhibited higher spectator medical demand per event, whereas MS-MSTs showed more distributed demand across multiple venues.

**Conclusion:**

This comparative, multi-event analysis provides evidence-based insights to inform scalable and effective medical staffing at major sporting tournaments. The findings demonstrate higher per-event utillization at SS-MSTs and distributed service requirements at MS-MSTs, offering practical guidance for optimizing resource allocation and medical planning strategies.

## Introduction

Medical planning for multi-day major sporting tournaments (MSTs) is a complex process encompassing a wide range of operational and clinical considerations (1–3). Medical preparedness at sporting venues is integrated into the emergency response frameworks of host cities (4–8). It requires close collaboration with local emergency medical services, referral hospitals, public health institutions, and regional crisis management centres (4–8). On-site, the venue medical team (VMT) operates in close cooperation with safety and security personnel, volunteers, and the event organisers (9, 10).

The composition, staffing levels, and equipment of the VMT are determined by multiple factors, including the type of sporting event, the characteristics and complexity of the venue, the requirements of the event organisers, anticipated attendance and time on site, spectator demographics, and prevailing weather conditions (6–11). Additional considerations–such as venue capacity, spatial layout, competition schedule, accreditation-based access control, and segmentation of spectator areas–also influence personnel deployment requirements (4, 7, 8, 10–12). The proximity of referral hospitals further affects the number of on-site ambulances required (13–15).

The objective of this study was to evaluate the scale of on-site medical interventions during major international sporting tournaments, based on data from two single-sport and two multi-sport MSTs. The findings from these MSTs contribute to evidence-based planning for future tournaments. The organization of four consecutive MSTs within the same medical system and reporting framework provided a unique opportunity to assess the differences among various types of tournaments.

## Materials and methods

This prospective study was designated to compare on-site medical interventions and hospital transports among spectators at venues during four major sporting tournaments of differing types: two single-sport (SS-MSTs)–The 2012 Final Tournament of the UEFA European Football Championship (UEFA EURO 2012) (16–18), The 2016 European Men’s Handball Championship (EHF EURO 2016) (19); and two multi-sport (MS-MSTs)–The World Games 2017 (TWG 2017) (20), The European Games 2023 (EG 2023) (21). All tournaments were conducted within consistent medical planning and healthcare system frameworks, with the exception of the UEFA EURO 2012 part in Ukraine; however its medical planning and reporting systems remained consistent.

1. Spectator population

A total of 2,188,497 spectators attended the tournaments: 1,853,588 (84.7%) during single-sport MSTs and estimated 334,909 (15.3%) during multi-sport MSTs. The SS-MSTs spectator numbers were based on reported actual attendance. The MS-MSTs estimated spectator numbers were based on ticket distribution. The demographics of the spectator population were obtained from official organiser reports (16, 19–21). Analyses related to demographic factors are based on these aggregate reports.

In total, 329 sporting events were analyzed, comprising 31 football matches, 48 handball matches during SS-MSTs, and 250 events across other sports during MS-MSTs. Overview of venues characteristics, disciplines, event numbers, and in-person spectator attendance is provided in the Supplementary materials.

1. Medical reporting framework

Data were collected using a standardized, anonymized daily medical reporting system. It was developed and supervised by the authors, who were responsible for medical coordination during the analyzed MSTs. For UEFA EURO 2012, the authors’ responsibilities were limited to the part of the tournament held in Poland; daily reports from Ukraine were obtained on the country level. Reports were submitted directly to the medical coordinator at the main operations centre of each tournament. The collected data were analyzed, aggregated into an MST database, and summarized in official reports.

1. Outcome measures

Using the reported daily medical data, this study primarily focused on comparisons of key metrics, including the number patient presentations (PP), patient presentation rates (PPR) per 10,000 spectator, the number of transports to hospital (TTH), transport-to-hospital rates (TTHR) per 10,000 spectators, and the medical-event-to-transport-to-hospital ratio (MEHR) across single- and multi-sport tournaments (22–27).

1. Data analysis

A prospective comparative approach was used to enable standardized, real-time data collection and minimize recall bias, with stated-preference elements incorporated to capture participant and venue-specific characteristics. Descriptive analyses included venue capacities, number of matches per venue, total attendance, and mean and median match attendance during SS-MSTs. For MS-MSTs, the analysis covered venues capacities, disciplines, number of sporting events, estimated total attendance, and mean attendance per sporting event.

Medical measures were summarized using the mean, median, standard deviation (SD), and 95% confidence intervals (CI). Independent proportions were compared using the z-test, with *p*-value < 0.05 considered statistically significant. To adjust for multiple comparisons and control type I error rate, the Bonferroni correction was applied. Data aggregation and descriptive statistics were performed in Microsoft Excel 2021 MSO, while inferential analyses were conducted in R 4.5.0.

## Results

1. Venues and spectators

- Single-sport tournaments

The UEFA EURO 2012 was held across eight host cities in Poland and Ukraine. It featured stadiums with seating capacities ranging from 35,000 to over 70,000 seats, and individual match attendance ranging from 31,840 to 64,640 (mean: 46,482). It attracted a total of 1,452,966 (66.4%) international spectators over the 24-day tournament.

The 2016 European Men’s Handball Championship (EHF EURO 2016) took place in four host cities in Poland, and utilized indoor arenas with seating capacities ranging from 6,500 to 15,000, and per-match figures from 6,000 to 15,000 (mean: 8,346). It drew a total of 400,622 (18.3%) international spectators during the 16-day tournament.

- Multi-sport tournaments

The World Games 2017 (TWG 2017) was a global, non-Olympic multi-sport event featuring 31 disciplines. This tournament was held across 26 different indoor and outdoor, sporting and temporary venues within a single host city. Venues capacities ranged from 500 to 42,000 seats. The estimated primarily local spectator population was 163,324 (7.5%), with mean of 1,420 spectators per event and the tournament lasted for 11 days.

The European Games 2023 (EG 2023) represented the third edition of this continental tournament. It featured a combination of 10 non-Olympic and 19 Olympic disciplines, and was conducted across 26 different indoor and outdoor, sporting and temporary venues in 14 host locations. Venues capacities ranged from as few as 100 seats to 54,378. The estimated mainly local spectator population was 171,585 (7.8%), with mean of 1,280 spectators per event, and the tournament lasted for 12 days.

Overall, spectator attendance at multi-sport tournaments was 5.5 times lower, whereas the number of sporting events was 3.1 times higher than at single-sport tournaments.

1. On-site patient presentations

At UEFA EURO 2012, a total of 1,285 on-site medical interventions (PP) were documented–662 in Poland (15 matches) and 623 in Ukraine (16 matches)–averaging 41 PP per match and 67 per match day. The mean PPR was 8.42, corresponding to one intervention per 1,131 spectators. EHF EURO 2016 recorded 124 on-site medical interventions, averaging 2.6 per match and 8.3 per match day. The mean PPR was 2.68, equivalent to one intervention per 3,231 spectators.

TWG 2017 recorded 104 on-site medical interventions, averaging of 0.96 per event or 9.4 per day. The mean PPR was 6.74, or one intervention per 1,570 spectators. EG 2023 recorded 26 on-site medical interventions, corresponding to 0.2 per event or 2.0 per day. The mean PPR was 1.62, equivalent to one intervention per 6,599 spectators.

A total of 1,539 (91.5%) on-site medical interventions were recorded during SS-MSTs and 130 (8.5%) during MS-MSTs. The mean PPR during SS-MSTs was 5.71 compared with 3.97 during MS-MSTs.

Overall, 0.07% of spectators required on-site medical interventions (0.07% at SS-MSTs and 0.04% at MS-MSTs). No spectator deaths or mass-casualty incidents occurred.

Daily on-site medical service data for spectators during MSTs are summarized in Tables 1 (SS-MSTs) and 2 (MS-MSTs).

1. Hospital transports

**Table 1:** Daily on-site patient presentations data for spectators during single-sporting major sport tournaments.

MST days	Spectators	Events	PP	PPR	Spectators/PP	PP/event
UEFA EURO 2012						
Day 1	97,070	2	138	14.21	703	69.0
Day 2	68,913	2	74	10.30	931	37.0
Day 3	78,700	2	47	5.97	1,674	23.5
Day 4	114,440	2	93	7.25	1,231	46.5
Day 5	96,920	2	113	11.66	856	56.5
Day 6	71,363	2	70	9.80	1,019	35.0
Day 7	78,450	2	43	5.48	1,824	21.5
Day 8	113,640	2	96	8.18	1,184	48.0
Day 9	97,070	2	135	13.90	719	67.5
Day 10	71,750	2	78	10.80	920	37.0
Day 11	77,520	2	63	8.10	1,230	31.5
Day 12	113,340	2	81	7.14	1,399	40.5
Day 14	55,950	1	47	8.40	1,190	47.0
Day 15	39,150	1	33	8.43	1,186	33.0
Day 16	48,000	1	16	3.33	3,000	16.0
Day 17	64,340	1	50	7.77	1,287	50.0
Day 20	48,000	1	26	5.41	1,846	26.0
Day 21	55,540	1	43	7.74	1,292	43.0
Day 24	63,170	1	39	6.17	1,620	39.0
Total	1,452,966	31	1,285	8.42	1,131	41.4
EHF EURO 2016						
Day 1	39,000	4	9	2.30	4,333	2.25
Day 2	28,472	4	12	4.21	2,373	3.00
Day 3	36,800	4	13	3.53	2,831	3.25
Day 4	26,960	4	13	4.82	2,074	3.25
Day 5	43,708	4	11	2.51	3,973	2.75
Day 6	29,722	4	12	4.03	2,477	3.00
Day 7	18,092	2	5	2.76	3,618	2.50
Day 8	12,000	2	6	5.00	2,000	3.00
Day 9	24,050	2	3	1.24	8,017	1.50
Day 10	13,000	2	3	2.30	4,333	1.50
Day 11	22,712	2	5	2.20	4,542	2.50
Day 12	13,000	2	3	2.30	4,333	1.50
Day 13	44,060	6	17	3.85	2,592	2.83
Day 15	34,046	4	9	2.64	3,783	2.25
Day 17	15,000	2	3	2.00	5,000	1.50
Total	400,622	48	124	2.68	3,231	2.58
SS-MST	1,853,588	79	1,409	5.71	1,316	17.8

MST, major sport tournament; PP, patient presentation; PPR, patient presentation rate per 10,000 spectators; SS-MST, single-sporting major sport tournament.

Days on which no matches were played during the tournament have not been included in the table.

**Table 2:** Daily on-site patient presentations data for spectators during multi-sporting major sport tournaments.

MST days	Spectators	Events	PP	PPR	Spectators/PP	PP/event
TWG 2017						
Day 1	12,500	1	13	10.40	8,654	13
Day 2	13,407	10	10	7.45	1,341	1
Day 3	26,635	15	15	5.63	1,776	1
Day 4	11,420	11	7	6.13	1,631	0.64
Day 5	14,251	10	4	2.81	3,563	0.4
Day 6	8,366	12	9	10.70	930	0.75
Day 7	11,182	12	10	8.94	1,118	0.83
Day 8	8,071	9	6	7.43	1,345	0.67
Day 9	13,901	10	5	3.59	2,780	0.5
Day 10	25,481	11	17	6.67	1,499	1.55
Day 11	18,110	7	8	4.42	2,264	1.14
Total	163,324	108	104	6.74	1,570	1.68
EG 2023						
Day-1	10,672	2	0	0	0	0
Day 1	9,360	8	5	5.34	1,872	0.63
Day 2	14,156	11	3	2.12	4,719	0.27
Day 3	13,274	13	3	2.26	4,425	0.23
Day 4	10,976	12	2	1.82	5,488	0.17
Day 5	24,380	15	6	2.46	4,063	0.4
Day 6	10,992	12	0	0	0	0
Day 7	20,098	14	1	0.99	20,098	0.07
Day 8	10,454	11	1	1.91	10,454	0.09
Day 9	12,656	10	0	0	0	0
Day 10	13,245	10	0	0	0	0
Day 11	15,284	12	4	2.61	3,821	0.33
Day 12	6,038	6	1	1.65	6,036	0.17
Total	171,585	136	26	1.62	6,599	0.19
MS-MST	334,909	244	130	3.97	2,576	0.52

MST, major sporting tournament; PP, patient presentation; PPR, patient presentation rate per 10,000 spectators; MS-MST, multi-sport major sporting tournament.

At UEFA EURO 2012, 111 hospital transports were recorded–50 in Poland and 61 in Ukraine–averaging 3.6 per match. The mean TTHR was 0.60, corresponding to one hospital transport per 13,090 spectators. The highest number of hospital transports during a single match was 10, with host city totals ranging from 3 to 30 across the tournament. The mean METH rate was 6.74.

During EHF EURO 2016, 11 hospital transports were recorded, averaging 0.2 per match. The mean TTHR was 0.18, equivalent to one hospital transport per 36,420 spectators. The mean METH rate was 5.18.

At TWG 2017 and EG 2023, 6 and 7 hospital transports were recorded, respectively, corresponding to one per 27,220 and one per 24,512 spectators. The mean TTHR was 0.58 for TWG 2017 and 0.39 for EG 2023. The corresponding mean METH rates were 6.88 and 14.10, respectively.

A total of 135 hospital transports were reported–122 during SS-MST and 13 during MS-MST. The mean TTHR during SS-MSTs was 0.42, compared with 0.47 during MS-MSTs. The overall mean METH rate was 8.0%, with 6.09% at SS-MSTs and 10.79% at MS-MSTs. Hospital transports during SS-MSTs accounted for the majority (90.3%) of all TTHs. Overall, 0.006% of spectators required hospital transports (0.006% at SS-MSTs and 0.004% at MS-MSTs).

Daily transport-to-hospital and medical-event-transport-to-hospital data for spectators during MSTs are presented in Tables 3 (SS-MSTs) and 4 (MS-MSTs).

1. Comparison of tournaments

- Tournament-level comparisons

**Table 3:** Daily transport-to-hospital and medical-event–transport-to-hospital data for spectators during single-sport major sporting tournaments.

MST days	Spectators	Events	TTH	TTHR	Spectators/TTH	TTH/event	METH
UEFA EURO 2012							
Day 1	97,070	2	7	0.70	13,867	3.5	4.82
Day 2	68,913	2	14	2.03	4,922	7.0	15.91
Day 3	78,700	2	4	0.50	19,675	2.0	7.84
Day 4	114,440	2	11	0.96	10,404	5.5	10.57
Day 5	96,920	2	4	0.41	24,230	2.0	3.42
Day 6	71,363	2	6	0.70	11,894	3.0	7.89
Day 7	78,450	2	5	0.63	15,690	2.5	10.41
Day 8	113,640	2	10	0.88	11,364	5.0	9.43
Day 9	97,070	2	12	1.23	8,089	6.0	8.16
Day 10	71,750	2	5	0.69	14,350	2.5	6.02
Day 11	77,520	2	5	0.64	15,504	2.5	7.35
Day 12	113,340	2	5	0.48	22,668	2.5	5.81
Day 14	55,950	1	3	0.71	18,650	3.0	6.00
Day 15	39,150	1	4	1.02	9,787	4.0	10.81
Day 16	48,000	1	3	0.62	16,000	3.0	15.79
Day 17	64,340	1	6	0.93	10,723	6.0	10.71
Day 20	48,000	1	6	0.20	8,000	6.0	18.75
Day 21	55,540	1	1	1.08	55,540	1.0	2.27
Day 24	63,170	1	0	0	0	0	0
Total	1,452,966	31	111	0.60	13,090	3.58	6.74
EHF EURO 2016							
Day 1	39,000	4	3	0.76	13,000	0.75	25.00
Day 2	28,472	4	3	1.05	9,491	0.75	20.00
Day 3	36,800	4	0	0	0	0	0
Day 4	26,960	4	2	0.74	13,480	0.50	13.30
Day 5	43,708	4	0	0	0	0	0
Day 6	29,722	4	0	0	0	0	0
Day 7	18,092	2	0	0	0	0	0
Day 8	12,000	2	1	0.08	12,000	0.50	14.28
Day 9	24,050	2	0	0	0	0	0
Day 10	13,000	2	0	0	0	0	0
Day 11	22,712	2	0	0	0	0	0
Day 12	13,000	2	0	0	0	0	0
Day 13	44,060	6	1	0.22	44,060	0.17	5.55
Day 15	34,046	4	1	0.29	34,046	0.25	10.00
Day 17	15,000	2	0	0	0	0	0
Total	400,622	48	11	0.18	36,420	0.23	5.18
SS-MST	1,853,588	79	122	0.42	15,193	1.54	6.09

MST, major sporting tournament; TTH, transport-to-hospital; TTHR, transport-to-hospital rate per 10,000 spectators; METH, medical-event–transport-to-hospital rate (%); SS-MST, single-sport major sporting tournament. Days on which no matches were played during the tournament have not been included in the table.

**Table 4:** Daily transport-to-hospital and medical-event–transport-to-hospital data for spectators during multi-sport major sporting tournaments.

MST days	Spectators	Events	TTH	TTHR	Spectators/TTH	TTH/event	METH
TWG 2017							
Day 1	12,500	1	0	0	0	0	0
Day 2	13,407	10	0	0	0	0	0
Day 3	26,635	15	0	0	0	0	0
Day 4	11,420	11	1	0.87	11,420	0.09	12.5
Day 5	14,251	10	1	0.70	14,251	0.1	20.00
Day 6	8,366	12	2	2.39	4,183	0.16	18.18
Day 7	11,182	12	0	0	0	0	0
Day 8	8,071	9	2	2.47	4,035	0.22	25.00
Day 9	13,901	10	0	0	0	0	0
Day 10	25,481	11	0	0	0	0	0
Day 11	18,110	7	0	0	0	0	0
Total	163,324	108	6	0.58	27,220	0.05	6.88
EG 2023							
Day −1	10,672	2	0	0	0	0	0
Day 1	9,360	8	0	0	0	0	0
Day 2	14,156	11	1	0.71	14,156	0.09	25.00
Day 3	13,274	13	1	0.75	13,274	0.07	25.00
Day 4	10,976	12	2	1.82	5,488	0.16	50.00
Day 5	24,380	15	0	0	0	0	0
Day 6	10,992	12	0	0	0	0	0
Day 7	20,098	14	1	0.50	20,098	0.07	50.00
Day 8	10,454	11	0	0	0	0	0
Day 9	12,656	10	0	0	0	0	0
Day 10	13,245	10	0	0	0	0	0
Day 11	15,284	12	2	1.31	7,642	0.16	33.33
Day 12	6,038	6	0	0	0	0	0
Total	171,585	136	7	0.39	24,512	0.05	14.10
MS-MST	334,909	244	13	0.48	25,762	0.05	10.80

MST, major sporting tournament; TTH, transport-to-hospital; TTHR, transport-to-hospital rate per 10,000 spectators; METH, medical-event–transport-to-hospital rate (%); MS-MST, multi-sport major sporting tournament.

Pairwise analyses of the four MSTs showed that UEFA EURO 2012 exhibited the highest mean on-site medical interventions ratio (PPR = 8.42), significantly higher than EHF EURO 2016 (PPR = 2.68), and EG 2023 (PPR = 1.62) after Bonferroni correction for six pairwise comparisons per outcome (adjusted *α* = 0.0083). On-site medical interventions ratio at TWG 2017 was significantly higher than at EG 2023–6.74 vs. 1.62, respectively.

Hospital transport ratio was significantly higher for UEFA EURO 2012 (TTHR = 0.60) compared with EHF EURO 2016 (TTHR = 0.18), and EG 2023 (TTHR = 0.39). Hospital transport ratio at TWG 2017 (TTHR = 0.58) differed only from EHF EURO 2016. For METH rates, only at EG 2023 (METH = 14.10%) was significantly higher than TWG 2017 (METH = 6.88%), while these rates do not differ significantly between UEFA EURO 2012, EHF EURO 2016, and TWG 2017 after Bonferroni correction.

Comparison of outcomes between tournaments are presented in Table 5.

- SS-MST vs. MS-MST comparisons

**Table 5:** Comparison of outcomes between tournaments.

Descriptive statistics	UEFA EURO 2012	EHF 2016	TWG 2017	EG 2023
Patient presentation (PP)				
Number	1,285	124	104	26
Mean	67.6	7.3	9.4	2
SD	35.0	5.1	4.1	2
95% CI	44.7–90.5	4.71–9.87	8.1–10.8	1.4–2.5
Patient presentation rate (PPR)^*^				
Mean	8.42	2.68	6.74	1.62
SD	2.82	1.45	2.6	1.50
95% CI	6.58–10.26	1.95–3.41	5.90–7.58	1.19–2.05
Transport to hospital (TTH)				
Number	111	11	6	7
Mean	4.6	0.64	0.5	0.5
SD	4.0	1.05	0.8	0.6
95% CI	3.0–7.2	0.11–1.17	0.3–0.7	0.4–0.6
Transport to hospital rate (TTHR)^*^				
Mean	0.6	0.18	0.58	0.39
SD	0.49	0.33	0.96	0.53
95% CI	0.28–0.92	0.02–0.34	0.27–0.89	0.24–0.54
Medical event to transport-to-hospital ratio (METH)^**^				
Mean	6.74	5.18	6.88	14.1
SD	5.48	8.21	9.95	19.94
95% CI	3.44–10.04	1.03–9.33	3.65–10.11	8.35–19.85

PPR: Significant pairwise (Bonf *α* ≈ 0.0083): UEFA EURO 2012 > EHF 2016, EG 2023.

TWG 2017 > EG 2023.

TTHR: Significant pairwise (Bonf *α* ≈ 0.0083): UEFA EURO 2012 > EHF EURO 2016, EG 2023.

TWG 2017 > EURO 2016.

METH: Significant pairwise (Bonf *α* ≈ 0.0083): EG 2023 > TWG 2017. UEFA EURO 2012, EHF EURO 2016, TWG 2017 comparable ^*^calculated per 10,000 spectators; ^**^percentage (%). SS-MST, single-sport major sporting tournament; MS-MST, multi-sport major sporting tournament.

When tournaments were compared by type, SS-MSTs had higher on-site medical interventions ratio compared with MS-MSTs (5.71 vs. 3.97) (*z* = 3.74, *p* < 0.001), which was statistically significant after Bonferroni correction for three outcomes (adjusted *α* = 0.0167). Differences in hospital transport ratios (0.42 vs. 0.48) (*z* ≈ 1.83, *p* ≈ 0.067) and rates of on-site medical interventions to hospital transports (6.09% vs. 10.80%) (*z* = 0.76, *p* ≈ 0.447) between SS-MSTs and MS-MSTs did not reach statistical significance after Bonferroni correction.

Comparison of outcomes between SS-MSTs vs. MS-MSTs are presented in Table 6.

**Table 6:** Comparison of outcomes between single-sport vs. multi-sport MSTs.

Descriptive statistics	SS-MST	MS-MST
Patient presentation (PP)		
Number	1,409	130
Mean	39.1	5.4
SD	39.7	4.9
95% CI	24.1–54.5	4.4–6.4
Patient presentation rate (PPR)^*^		
Mean	5.71	3.97
SD	3.67	3.29
95% CI	4.30–7.21	3.27–4.67
*z*-score	3.74 p < 0.001	
Significant (Bonf *α* ≈ 0.0167)	Yes	
Transport to hospital (TTH)		
Number	122	13
Mean	2.9	0.5
SD	3.6	0.7
95% CI	1.5–5.3	0.4–0.6
Transport to hospital rate (TTHR)^*^		
Mean	0.42	0.48
SD	0.47	0.75
95% CI	0.24–0.60	0.32–0.62
*z*-score	*z* ≈ 1.83, *p* ≈ 0.067	
Significant (Bonf *α* ≈ 0.0167)	No	
Medical event to transport-to-hospital ratio (METH)^**^		
mean	6.09	10.80
SD	6.7	16.25
95% CI	3.52–8.66	7.33–14.25
*z*-score	*z* = 0.76, *p* ≈ 0.447	
Significant (Bonf *α* ≈ 0.0167)	no	

^*^Calculated per 10,000 spectators; ^**^percentage (%). SS-MST, single-sport major sporting tournament; MS-MST, multi-sport major sporting tournament.

## Discussion

This study provides a comprehensive analysis of on-site medical service utilization during four major international sporting tournaments, comparing two single-sport and two multi-sport tournaments (16–21). The findings highlight key differences in attendance patterns, patient presentations, hospital transport, and medical workload, revealing important considerations for MSTs planning, resource allocation, and public health preparedness (2, 6–8, 10, 11, 24, 27–37).

1. Spectator attendance and tournament characteristics

Single-sport tournaments attracted significantly higher spectator numbers, accounting for 84.7% of total attendance. In contrast, multi-sport tournaments consisted of a greater number of smaller, dispersed sporting events, resulting in lower overall attendance. Notably, these characteristics of MS-MSTs reduce the risk of mass-casualty incidents but increase the logistical challenges of maintaining adequate on-site medical coverage across multiple venues simultaneously (10, 12, 13, 24, 27–29, 33, 38–45).

Although UEFA EURO 2012 was co-hosted by Poland and Ukraine and therefore operated within two distinct healthcare systems, consistency in medical planning and reporting was ensured by the event organizer (22, 23, 26). All other tournaments were held entirely in Poland within the framework of European Union healthcare policies.

Spectators demographics differed between tournaments (24, 27, 28). SS-MSTs attracted a high proportion of international spectators due to ticket distribution through sports federations, resulting in demand for tickets that substantially exceeded their availability. The higher proportion of non-local attendees involved in different tournament-related activities influenced both the volume and nature of medical presentations (7, 9, 10, 12, 14, 27, 34, 46–52).

In contrast, MS-MSTs primarily attracted local residents, resulting in a more dispersed and heterogeneous audience whose involvement was mostly limited to the sporting events (38, 39, 53, 54). Attendance figures for MS-MSTs—particularly EG 2023—may have been overestimated, as they were calculated based on ticket distribution rather than verified attendance.

1. On-site patient presentations

On-site patient presentations varied substantially across analyzed MSTs. Overall, the medical services were utilized at a low rate, with only 0.07% of attendees requiring medical care at SS-MSTs and 0.04% at MS-MSTs.

UEFA EURO 2012, the largest of the analyzed MSTs, recorded the highest mean on-site medical intervention ratio. This is likely attributable to the combined effects of high crowd density–mean match attendance 46,870 per venue, 5.6 times higher than at EHF EURO 2016–diverse spectator demographics and tournament intensity. Notably, the number of on-site medical interventions ranged from 16 to 69 per match—or one intervention per 703–3,000 spectators—providing a basis for evidence-based planning of venue medical team resources.

EHF EURO 2016 showed a markedly lower burden on on-site medical services, reflecting lower crowd density and smaller sport venue capacities. TWG 2017 demonstrated relatively higher medical demand, potentially related to the dispersed nature of the sporting events, which were often held in temporary facilities, as well as the inclusion of several high–attendance events, such as speedway, American football, and opening or closing ceremonies. EG 2023 exhibited the lowest PPR, likely due to smaller crowds and attendees demographics; per-event attendance was based on ticket distribution, which may slightly overestimate actual numbers, but still allows for comparisons across events.

This pattern aligned with previous research indicating that medical workload correlates more closely with crowd density and event type than with absolute attendance (27, 45–50, 52–56). In the previous reports, the recorded on-site medical intervention ratios during SS-MSTs (PPR = 0.1–4.5) were comparable to those during MS-MSTs (PPR = 0.2–4.5) (30, 45–50). For example, the UEFA Under-21 Championship 2019 in Italy recorded 31 on-site medical interventions across four matches attended by 72,655 spectators (PPR = 4.1) (46). The 2019 Rugby World Cup in Japan reported 449 on-site medical interventions over 45 matches with 1.7 million spectators (PPR = 2.6) (47). Non-tournament data reflect similar trends: at King Baudouin Stadium in Brussels (2010–2019), 626 PPs were recorded over 41 matches attended by 1.63 million spectators (PPR = 3.8) (30). However, during highly crowded tournaments, such as the 2014 FIFA World Cup in Brazil, 6,222 on-site medical interventions were reported among over 3.4 million spectators (PPR = 18.3) (48).

MS-MSTs, by contrast, displayed greater variability. At the 2006 Commonwealth Games in Melbourne, with 1.72 million spectators across 21 venues over 12 days, 2,279 on-site medical interventions were reported (PPR = 1.32) (45). The 2005 Athletics World Championships in Helsinki recorded 1,586 on-site medical interventions among 479,000 spectators (PPR = 0.2) (38), the 1999 Athletics Championships in Seville had 1,338 on-site medical interventions with nearly 0.5 million attendees (PPR = 4.5) (39), and the 1988 Winter Olympics in Calgary reported 796 on-site medical interventions among 1.8 million spectators (PPR = 4.42) (40).

1. Hospital transports

Hospital transport rates during tournaments were correspondingly low, representing 0.006% of spectators at SS-MSTs and 0.004% at MS-MSTs––approximately 10 times lower than overall on-site medical intervention ratios. The highest total hospital transport ratio was recorded at UEFA EURO 2012, reflecting both higher ratio of on-site medical interventions and the large absolute spectator numbers. MS-MSTs demonstrated lower hospital transport ratios, but occasionally higher mean hospital transport to medical interventions rate values, as observed at the EG 2023, likely due to the low absolute numbers of medical workload and the audience demographics at the MS-MSTs.

The approximately tenfold difference between on-site medical interventions and hospital transport ratios was consistent with other MSTs–for example, UEFA Under-21 Championship 2019 in Italy (PPR = 4.1 vs. TTHR = 0.4), 2019 Rugby World Cup in Japan (PPR = 2.6 vs. TTHR = 0.2), 41 matches at King Baudouin Stadium (PPR = 3.9 vs. TTHR = 0.4), and others (30, 45–47, 53, 54, 56–62).

1. Comparison of tournaments

Statistical analyses confirmed that the largest MST–UEFA EURO 2012–exhibited the highest ratios of on-site medical services and hospital transports (PPR and TTHR, respectively). Higher-attendance sporting events during TWG 2017 may also have contributed to the increased on-site medical workload and hospital transports. However, the proportion of medical conditions requiring hospital transports was comparable across the three tournaments. The relatively high METH observed at EG 2023 may have been influenced by the low absolute numbers of on-site medical services and hospital transports recorded during the tournament.

Comparison of single- and multi-sport tournaments revealed significantly higher ratio of on-site medical services (PPR) at SS-MSTs, consistent with previous analyses. Transport to hospital ratio (TTHR) and METH values were similar, indicating a consistent profile of mild and severe medical conditions across both tournament types.

1. Implications for medical preparedness and event management

The findings provide strong support for evidence-based medical planning for upcoming MSTs, emphasizing the need for flexible, scalable medical resources tailored to tournament type, crowd size, and audience demographics (1, 3, 5, 7, 10, 13, 27).

For single-sport tournaments, concentrated spectator populations during high-attendance sporting events result in higher per-event medical presentations rates. These require adequate on-site medical teams positioned strategically around venues, supported by rapid triage systems and efficient hospital transport pathways (9, 13, 14, 17, 47–50, 53, 55).

For multi-sport tournaments, although individual events may generate lower spectator medical burdens, the dispersed nature of these tournaments–often involving a larger number of venues–imposes distinct medical demands. Event organisers should therefore ensure integrated medical planning that balances on-site resources, inter-venue medical cooperation, preparedness for hospital transports, and established cooperation with local public health authorities (12, 38–40, 43–45, 54).

Variation in per-event medical rates further highlights the importance of real-time monitoring and dynamic adjustment of staffing throughout tournaments. Effective coverage requires distributed deployment of medical teams, supported by a centralized coordination system capable of managing multiple venues simultaneously.

1. Strengths and limitations

Strengths of this study include the large overall sample size, detailed tournament medical data, and a consistent healthcare system environment, which enabled direct comparison across tournaments. Additionally, the inclusion of MSTs from Central and Eastern Europe also helps address a gap in the current literature.

Limitations include reliance on estimated spectator numbers for multi-sport tournaments. In addition, detailed individual-level demographic data were not available, which limits the scope of analyses involving spectator characteristics. Despite this constraint, the findings provide a robust foundation for evidence-based medical planning for major sporting tournaments.

## Conclusion

Single-sport tournaments attracted larger, concentrated crowds, resulting in higher per-event medical presentations and hospital transport requirements among spectators, although overall rates of medical intervention remained low. Multi-sport tournaments generated lower per-event medical workload, but required precise medical planning due to the number of events. Across all tournaments, fewer than 10 % of on-site medical interventions required hospital transport, underscoring the effectiveness of on-site medical care.

These findings should be interpreted with consideration of the key limitation associated with estimated attendance at multi-sport tournaments. Nevertheless, the results support evidence-based medical planning.

Medical demand at both SS- and MS-MSTs can be effectively addressed through adaptable, venue-specific staffing, and coordinated inter-venue protocols. SS-MSTs require concentrated on-site resources and efficient triage and transport pathways, whereas MS-MSTs benefit from centralized coordination, dynamic staff allocation, and integrated multi-venue planning. Overall, these insights support scalable and context-sensitive strategies for optimizing medical services at major sporting tournaments.

## Data Availability

The raw data supporting the conclusions of this article will be made available by the authors, without undue reservation.

## References

[1] Hendricks S Rotunno A Gordon L Ganda J Zondi PC Derman W . Mass-gathering in sport: medicine, leadership and mentorship. Br J Sports Med. (2024) 58:528–30. doi: 10.1136/bjsports-2024-108377 38580399

[2] World Health Organization. Public Health for Mass Gatherings: Key Considerations. Geneva: World Health Organization (2015).

[3] Suy K Gijsenbergh F Baute L. Emergency medical assistance during a mass gathering. Eur J Emerg Med. (1999) 6:249–54. doi: 10.1097/00063110-199909000-00014, 10622392

[4] Khan AA Sabbagh AY Ranse J. Mass gathering medicine in soccer leagues: a review and creation of the SALEM tool. Int J Environ Res Public Health. (2021) 18:9973. doi: 10.3390/ijerph18199973 34639274 PMC8508246

[5] Stiers M Van Asbroeck PJ Hoogmartens O Guldentops J Sabbe M. Redefining the role of emergency medicine in mass gatherings. Eur J Emer Med. (2024) 31:171–2. doi: 10.1097/MEJ.0000000000001131, 38411506

[6] Schwartz B Nafziger S Milsten A Luk J Yancey A. Mass gathering medical care: resource document for the National Association of EMS physicians position statement. Prehosp Emerg Care. (2015) 19:559–68. doi: 10.3109/10903127.2015.1051680 26270473

[7] Locoh-Donou S Yan G Berry T O’Connor R Sochor M Charlton N . Mass gathering medicine: event factors predicting patient presentation rates. Intern Emerg Med. (2016) 11:745–52. doi: 10.1007/s11739-015-1387-1, 26758062

[8] Margolis AM Leung AK Friedman MS McMullen SP Guyette FX Woltman N. Position statement: mass gathering medical care. Prehosp Emerg Care. (2021) 25:593–5. doi: 10.1080/10903127.2021.1903632 33886431

[9] Roberts DM Blackwell TH Marx JA. Emergency medical care for spectators attending national football league games. Prehosp Emerg Care. 1:149–55. doi: 10.1080/10903129708958809 9709358

[10] Koski A Kouvonen A Sumanen H. Preparedness for mass gatherings: factors to consider according to the rescue authorities. Int J Env Res Pub H. (2020) 17:1361. doi: 10.3390/ijerph17041361, 32093217 PMC7068565

[11] Schwartz B Nafziger S Milsten A Luk J Yancey A 2nd. 2nd. Mass gathering medical care: resource document for the National Association of EMS physicians position statement. Prehosp Emerg Care. (2015) 19:559–68. 26270473

[12] Piat SC Minniti D Traversi D Gianino MM Massazza G Siliquini R. Torino 2006 winter Olympic games: highlight on health services organization. J Emerg Med. (2010) 39:454–61. doi: 10.1016/j.jemermed.2009.08.028 19879085

[13] Simon E Trager JD Wilson B. "Patient care at mass gathering events: basic, advanced, and critical care". In: Brady WJ Sochor MR Pepe PE Maino JC II Dyer KS, editors. Mass Gathering Medicine: A Guide to the Medical Management of Large Events. Cambridge: Cambridge University Press (2024). p. 6–20.

[14] Roberts DM Blackwell TH Marx JA. Emergency medical care for spectators attending national football league games. Prehosp Emerg Care. (1997) 1:149–55. doi: 10.1080/10903129708958809, 9709358

[15] Sekendiz B. Incidence, bystander emergency response management and outcomes of the out-of-hospital cardiac arrest at exercise and sport facilities in Australia. Emerg Med Austalas. (2021) 33:100–6. doi: 10.1111/1742-6723.13595, 32869475

[16] UEFA EURO. Social Responsibility Report. Madrid: UEFA EURO (2012).

[17] Smallwood CAH Arbuthnott KG Banczak-Mysiak B Borodina M Coutinho AP Payne-Hallström L . Euro 2012 European football championship finals: planning for a health legacy. Lancet. (2014) 383:2090–7. doi: 10.1016/S0140-6736(13)62384-3 24857705

[18] Boratyński J Borowski J Czerniak A Plich M. Aktualizacja Raportu na temat wpływu przygotowań i organizacji Mistrzostw Europy w Piłce Nożnej UEFA EURO 2012TM na gospodarkę Polski. Madrid: UEFA EURO.

[19] European Handball Federation. EHF Sport Report 2016. Vienna: European Handball Federation (2016). p. 10–3.

[20] The World Games. Wrocław 2017 – Final Report. Available online at: https://www.theworldgames.org/files/wg2017/2017-12-29%20WOC%20FINAL%20REPORT.pdf (Accessed March 25, 2026).

[21] Mierzwa P Bugajska M Raniszewski S Borek D Dróżdż M Tomanek M. Management of the European games 2023 by public administration - organizational and legal aspects. J Educ Health Sport. (2023) 41:83–107. doi: 10.12775/JEHS.2023.41.01.007

[22] Çalışkan C Kuday AD Özcan T Dağ N Kınık K. Quantitative metrics in mass-gathering studies: a comprehensive systematic review. Prehosp Disaster Med. (2024) 39:195–205. doi: 10.1017/S1049023X2400027X, 38576262

[23] Lund A Turris SA Amiri N Lewis K Carson M. Mass-gathering medicine: creation of an online event and patient registry. Prehosp Disaster Med. (2012) 27:601–11. doi: 10.1017/S1049023X12001367, 23031486

[24] Zeitz KM Zeitz CJ Arbon P. Forecasting medical work at mass-gathering events: predictive model versus retrospective review. Prehosp Disaster Med. (2005) 20:164–8. doi: 10.1017/s1049023x00002399, 16018504

[25] Hartman N Williamson A Sojka B Alibertis K Sidebottom M Berry T . Predicting resource use at mass gatherings using a simplified stratification scoring model. Am J Emerg Med. (2009) 27:337–43. doi: 10.1016/j.ajem.2008.03.042, 19328380

[26] Ranse J Hutton A. Minimum data set for mass-gathering health research and evaluation: a discussion paper. Prehosp Disaster Med. (2012) 27:543–50. doi: 10.1017/S1049023X12001288 23174040

[27] Sultana N Crilly J Ware RS Ranse J. Factors influencing patient presentation and transfer to hospital rates during mass-Gathering Stadium events: a scoping review. Prehosp Disaster Med. (2025) 40:101–13. doi: 10.1017/S1049023X25000287, 40207555 PMC12018010

[28] Locoh-Donau S Guofen Y Welcher M Berry T O’Connor RE Brady WJ . Mass-gathering medicine: a descriptive analysis of a range of mass-gathering event types. Am J Em Med. (2013) 31:843–6. doi: 10.1016/j.ajem.2013.01.016, 23453125

[29] Ranse J Hutton A Keene T Lenson S Luther M Bost N . Health service impact from mass gatherings: a systematic literature review. Prehosp Disaster Med. (2017) 32:71–7. doi: 10.1017/S1049023X16001199, 27938460

[30] Spaepen K Lauwaert D Kaufman L Haenen WAP Hubloue I. Validation of a Belgian prediction model for patient encounters at football mass gatherings. Prehosp Disaster Med. (2021) 36:724–9. doi: 10.1017/S1049023X21000923, 34538289

[31] Leusveld E Kleijn S Umans V. Usefulness of emergency medical teams in sport stadiums. Am J Cardiol. (2008) 101:712–4. doi: 10.1016/j.amjcard.2007.10.040, 18308027

[32] Northwest Center for Public Health Preparedness. Mass Gatherings: Are You Prepared?. Available online at: www.nwcphp.org/docs/mass_gatherings/mass_gathering_print_version.pdf (Accessed August 8, 2025).

[33] Peters GA Goralnick E. "Occurrence of the mass casualty incident at a mass gathering event". In: Brady WJ Sochor MR Pepe PE Maino JC II Dyer KS, editors. Mass Gathering Medicine: A Guide to the Medical Management of Large Events. Cambridge: Cambridge University Press (2024). p. 320–41.

[34] Arbon P Bridgewater FHG Smith C. Mass gathering medicine: a predictive model for patient presentation and transport rates. Prehosp Disaster Med. (2001) 16:150–8. doi: 10.1017/S1049023X00025905, 11875799

[35] Turris SA Lund A. "Principles of mass gathering health". In: Koenig KL Schultz CH, editors. Disaster Medicine: Comprehensive Principles and Practices. Cambridge: Cambridge University Press (2016). p. 585–602.

[36] Kruk ME Myers M Varpilah ST Dahn BT. What is a resilient health system? Lessons from Ebola. Lancet. (2015) 385:1910–2. doi: 10.1016/S0140-6736(15)60755-3, 25987159

[37] Memish ZA Steffen R White P Dar O Azhar EI Sharma A . Mass gathering medicine: public health issues arising from mass gathering religious and sporting events. Lancet. (2019) 393:2073–84. doi: 10.1016/S0140-6736(19)30501-X, 31106753 PMC7159069

[38] Hiltunen T Kuisma M Määttä T Tennilä A Hari T Bäckman R . Prehospital emergency care and medical preparedness for the 2005 world championship games in athletics in Helsinki. Prehosp Disaster Med. (2007) 22:304–11. doi: 10.1017/S1049023X0000491X, 18019097

[39] Millán EM Bonilla F Alonso JM Casado F. Medical care at the VIIth international amateur athletics federation world championships in athletics ‘Sevilla ‘99’. Eur J Emerg Med. (2004) 11:39–43. doi: 10.1097/00063110-200402000-00008, 15167192

[40] Thompson JM Savoia G Powell G Challis EB Law P. Level of medical care required for mass gatherings: the XV winter Olympic games in Calgary. Can Ann Emerg Med. (1991) 20:385–90. doi: 10.1016/S0196-0644(05)81660-9, 2003667

[41] Nakaze S Nakagawa K Tanaka S Homma Y Tanaka H. Epidemiology of emergency transport at sports stadiums and mass gatherings. J EMS Med. (2024) 3:1–10. doi: 10.35616/jemsm.2023.00045

[42] Craven P Hansroth J Quedado KD Goode CS Dragan S Monseau A . Lessons on mass gatherings learned from the 2019 union Cycliste Internationale Mountain bike world cup. Cureus. (2021) 13:e14275. doi: 10.7759/cureus.14275, 33954076 PMC8092131

[43] Enock KE Jacobs J. The Olympic and Paralympic games 2012: public health planning and operational challenges. Public Health. (2008) 122:1229–38. doi: 10.1016/j.puhe.2008.04.016, 18619630

[44] Public Health England. London 2012 Olympic and Paralympic Games: Summary Report (published 25 Jan 2013). Available online at: https://www.gov.uk/government/publications/london-2012-olympic-and-paralympic-games-summary-report#:~:text=Details.%20The%20Health%20Protection%20Agency%2C%20now%20Public,during%20the%20Olympic%20and%20Paralympic%20Games%20including (Accessed March 27, 2026).

[45] Dutch MJ Senin LM Taylor DJ. Mass gathering medicine: the Melbourne 2006 commonwealth games experience. Emerg Med Australasia. (2008) 20:228–33. doi: 10.1111/j.1742-6723.2008.01085.x, 18462409

[46] Imbriaco G Flauto A Bussolari T Cordenons F Gordini G. Mass gathering emergency medicine Organization for the Union of European football associations’ Under-21 championship 2019 in Bologna, Italy. Disaster Med Public Health Prep. (2020) 16:405–8. doi: 10.1017/dmp.2020.291, 33023705

[47] Tajima T Takazawa Y Yamada M Moriya T Sato H Higashihara J . Spectator medicine at an international mega sports event: Rugby world cup 2019 in Japan. Environ Health Prev Med. (2020) 25:72. doi: 10.1186/s12199-020-00914-0, 33234126 PMC7684143

[48] Arliani GG Lara PHS Pedrinelli A Ejnisman B Leite LMB Cohen M . Analysis of medical assistance provided to spectators at the 2014 FIFA world cup matches. Acta Ortop Bras. (2018) 26:33–5. doi: 10.1590/1413-785220182601178667, 29977142 PMC6025498

[49] Hardcastle TC Samlal S Naidoo R Hendrikse S Gloster A Ramlal M . A redundant resource: a pre-planned casualty clearing station for a FIFA 2010 stadium in Durban. Prehosp Disaster Med. (2012) 27:409–15. doi: 10.1017/S1049023X12000453, 22591650

[50] Goldberg SA Battistini V Cash RE Kelleher M Laporte C Peters G . A retrospective review of out of hospital cardiac arrest at Gillette Stadium: 10 years of experience at a large sports venue. Resusc Plus. (2023) 14:100386. doi: 10.1016/j.resplu.2023.100386, 37056959 PMC10085775

[51] Grima RS Carreño MJ Abadal LT Brossa V Ligero C Pons J . Acute coronary events among spectators in a soccer stadium. Rev Esp Cardiol. (2005) 58:587–91. 15899201

[52] Crawford M Donnelly J Gordon J MacCallum R MacDonald I McNeill M . An analysis of consultation with the crowd doctors at Glasgow Celtic football club, season 1999-2000. Br J Sport Med. (2001) 35:245–9. doi: 10.1136/bjsm.35.4.245, 11477019 PMC1724346

[53] Mercalli C Ghio FE Bonizzato S Mantellini B Serini C Pallavicini P . Providing medical care at mass gathering sporting events: the 2023 Ryder cup experience. BMC Emerg Med. (2025) 25:151. doi: 10.1186/s12873-025-01316-7, 40796818 PMC12345102

[54] Li Z Li S Liu Z Li S Huang H Wang T . Sports injury and illness incidence at the 2021 summer Universiade: a retrospective study of 6,500 athletes from 113 countries/regions. Front Public Health. (2025) 13:1532849. doi: 10.3389/fpubh.2025.1532849, 40371277 PMC12075538

[55] Khurana B Prakash J Chopra RR Loder RT. Effect of the NFL'S super bowl on emergency department visits for assault-related injuries. Emerg Radiol. (2024) 31:7–16. doi: 10.1007/s10140-023-02188-9 38012430 PMC11175618

[56] Pratt A Sale T Sanau E Ramo J Phillips G. Impact of the XVII Pacific games on the National Referral Hospital Emergency Department, Solomon Islands. Emerg Med Australas. (2025) 37:e14548. doi: 10.1111/1742-6723.14548, 39744771

[57] Spaepen K Cardinas R Haenen WAP Kaufman L Hubloue I. The impact of in-event health Services at Europe's largest electronic dance music festival on ems and Ed in the host community. Int J Environ Res Public Health. (2023) 20:3207. doi: 10.3390/ijerph20043207, 36833901 PMC9962375

[58] Lipska E Mikrut M Pawlak A Kavouras A. Emergency medical conditions requiring hospital transport during major sport tournaments: a retrospective analysis. Emerg. Med Serv. (2026) 13:11–6. doi: 10.36740/EMEMS202601102

[59] PoshtMashhadi A Wood LC Maghsoodi AI. The impact of mass gatherings and events on emergency department admissions: a systematic literature review. J Emerg Med. (2025) 74:23–48. doi: 10.1016/j.jemermed.2025.02.010, 40393823

[60] Qiu YS Crilly J Zimmerman PA Ranse J. The impact of mass gatherings on emergency department patient presentations with communicable diseases related to syndromic indicators: an integrative review. Prehosp Disaster Med. (2020) 35:206–11. doi: 10.1017/S1049023X20000151, 32070453

[61] Lipska E Kavouras A. Impact of major international sport tournaments on the local healthcare system in Poland: a retrospective analysis. Med Res J. (2026) 11:e04426005. doi: 10.5603/mrj.110724

[62] Boyle J Jessup M Crilly J Green D Lind J Wallis M . Predicting emergency department admissions. Emerg Med J. (2012) 29:358–65. doi: 10.1136/emj.2010.103531 21705374

