# Supplementary material for: On-site medical interventions for spectators at venues during major international sporting tournaments: a prospective comparative study of four single-sport and multi-sport tournaments
Source: Front Public Health. 2026 May 7;14:1812763. doi: 10.3389/fpubh.2026.1812763 (PMC13190376; doi:10.3389/fpubh.2026.1812763)
Supplement: Supplementary file 1 [file Table_1.DOCX]

Supplementary Digital Content

Overview of venues characteristics, disciplines, event numbers, and in-person spectator attendance at single- and multi-sport tournaments

1. Single-Sport Tournaments

| *venue* | *capacity* | *discipline* | *sporting event* | *total attendance* | *mean match attendance* | *median match attendance* |
| --- | --- | --- | --- | --- | --- | --- |
| UEFA EURO 2012 | | | | | | |
| National Stadium, Warsaw | 58,580 | Football | 5 | 278,710 | 55,742 | 55,590 |
| Wrocław Stadium | 45,105 |  | 3 | 123,480 | 41,160 | 41,000 |
| Metalist Stadium, Kharkiv | 40,003 |  | 3 | 111,118 | 37,632 | 38,373 |
| Arena Lviv | 34,915 |  | 3 | 99,130 | 33,043 | 32,990 |
| Gdańsk Stadium | 43,615 |  | 4 | 117,750 | 39,250 | 39,150 |
| Poznań Stadium | 43,269 |  | 3 | 116,920 | 38,973 | 39,150 |
| Donbas Arena, Donetsk | 51,504 |  | 5 | 238,500 | 48,700 | 48,700 |
| Olympic Stadium, Kyiv | 70,050 |  | 5 | 319,450 | 64,268 | 64,600 |
| Total |  |  | **31** | **1,452,966** | **46,870** | **41,480** |
| EHF EURO 2016 | | | | | | |
| Kraków Arena | 14,999 | Handball | 19 | 231,650 | 12,192 | 14,546 |
| Spodek Arena, Katowice | 9,803 |  | 6 | 34,800 | 5,800 | 7,000 |
| Centennial Hall, Wrocław | 6,500 |  | 17 | 98,068 | 5,769 | 6,500 |
| Ergo Arena, Gdańsk | 10,000 |  | 6 | 36,104 | 6,017 | 7,952 |
| Total |  |  | **48** | **400,622** | **8,346** | **7,476** |

2. Multi-Sport Tournaments

| *venue* | *capacity* | *discipline* | | *sporting events* | *estimated total attendance* | *estimated mean event attendance* |
| --- | --- | --- | --- | --- | --- | --- |
| The World Games 2017 (Wrocław) | | | | | | |
| Sport Hall | 500 | Archery | | 8 | 1,140 | 142 |
| Congress Centre | 550 | Billiards | | 5 | 2,050 | 410 |
| Congress Hall | 6 000 | Dance Sport | | 2 | 6,447 | 3,223 |
|  |  | Gymnastics | | 13 | 12,913 | 993 |
| Animals Park | 500 | Orienteering | | 3 | 1,566 | 522 |
| Stadium | 1,500 | Fistball | | 4 | 3,166 | 791 |
|  |  | Lacrosse | | 4 | 4,125 | 1,031 |
| Market Square | 1,500 | Sport climbing | | 3 | 5,604 | 1,868 |
| Forum of Music | 1,500 | Powerlifting | | 3 | 1,977 | 659 |
| Open Fields | 1,900 | Beach Handball | | 4 | 7,856 | 1,964 |
| Olympic Stadium | 11,000 | American Football | | 2 | 18,418 | 9,209 |
|  |  | Speedway | | 1 | 11,536 | 11,536 |
| Multifunctional Hall | 1,300 | Tug of War | | 3 | 1,269 | 423 |
| Sports Complex | 900 | Indoor rowing | | 2 | 643 | 321 |
| Sports Complex | 1,200 | Ju-Jitsu | | 2 | 2,288 | 1,144 |
|  |  | Karate | | 2 | 3,917 | 1,958 |
|  | 1,500 | Flying Disc | | 3 | 7,874 | 2,625 |
| Sport Centre | 1,200 | Uni-hockey | | 5 | 3,567 | 713 |
|  |  | Korfball | | 5 | 5,040 | 1,008 |
| Pergola | 500 | Boulles | | 4 | 1,311 | 328 |
| Sky Tower | 130 | Bowling | | 4 | 400 | 100 |
| City Rink | 1,700 | Roller Sports | | 5 | 11,244 | 2,249 |
| Squash Centre | 500 | Squash | | 4 | 1,515 | 379 |
| Airport | 1,000 | Air Sports | | 4 | 2,488 | 622 |
| Stadium | 42,000 | Ceremonies | | 2 | 25,501 | 12,750 |
| Swimming Pools | 1,000 | Canoe polo | | 3 | 2,851 | 950 |
|  | 700 | Fin-swimming | | 2 | 1,578 | 394 |
|  | 700 | Life-saving | | 2 |  | 394 |
| Sport Hall | 3,000 | Muaythai | | 2 | 5,294 | 2,647 |
|  |  | Kickboxing | | 2 | 4,539 | 2,269 |
|  |  | Sumo | | 2 | 3,407 | 1,703 |
| River Marina | 1,000 | Waterski & Wakeboard | | 5 | 1,800 | 360 |
| Total | 199,507* |  | | **116** | **163,324**** |  |
| European Games 2023 (Kraków and Małopolska Region) | | | | | | |
| Sport Arena | 3,000 | Karate | | 2 | 4,596 | 2,298 |
| Stadium | 54,378 | Athletics | | 6 | 21,233 | 3,538 |
| Sport Arena | 600 | Table Tennis | | 9 | 4,136 | 460 |
| BMX Park | 2,300 | Cycling BMX Freestyle | | 2 | 3,524 | 1,762 |
| Sports Centre | 1,200 | Canoe Slalom | | 4 | 3,677 | 919 |
| Archery Park | 500 | Archery | | 7 | 2,681 | 383 |
| Sport Center | 2,686 | Modern Pentathlon | | 6 | 11,345 | 2,057 |
| Sport Arena | 1,000 | 3x3 Basketball | | 4 | 3,064 | 766 |
| Sport Hall | 2,500 | Fencing | | 6 | 11,490 | 1,915 |
| Stadium | 4,000 | Rugby Sevens | | 3 | 8,192 | 3,064 |
| Park and Lake | 1,000 | Triathlon | | 3 | 2,298 | 766 |
| City Square | 1,000 | Teqball | | 4 | 3,064 | 766 |
| Sport Centre | 1,000 | Padel | | 5 | 3,830 | 766 |
| Waterway | 1,200 | Canoe Sprint | | 4 | 3,677 | 919 |
| Sport Arena | 560 | Kickboxing | | 3 | 4,386 | 731 |
|  |  | Muaythai | | 3 |  | 731 |
| Hill Park | 9,500 | Cycling Mountain Bike | | 1 | 7,277 | 7,277 |
| City Park | 2,700 | Breaking | | 2 | 4,136 | 2,068 |
| Sport Hall | 1,500 | Taekwondo | | 4 | 3,213 | 803 |
| Ice Hall | 1,500 | Judo | | 1 | 1,149 | 1,149 |
| Aquatic Centre | 100 | Artistic swimming | | 5 | 383 | 77 |
| Diving Arena | 320 | Diving | | 7 | 1,400 | 200 |
| Sport Arena | 1,000 | Boxing | | 9 | 6,624 | 766 |
| Sport Arena | 1,600 | Badminton | | 7 | 7,579 | 1,226 |
| Beach Arena | 2,000 | Beach Handball | | 3 | 4,596 | 1,532 |
|  |  | Beach Soccer | | 5 | 7,660 | 1,532 |
| Climbing Centre | 2,500 | Sport Climbing | | 4 | 7,650 | 1,915 |
| Shooting Centre | 500 | Shooting | | 11 | 4,213 | 383 |
| Ski Jumping Hills | 8,000 | Ski Jumping | | 4 | 25,512 | 4,547 |
| Total | 223,623* |  |  | **134** | **171,585**** | **1,262** |

*total number of tickets; **distributed tickets
